# Advance in integrating platinum-based chemotherapy with radiotherapy for locally advanced nasopharyngeal carcinoma

**DOI:** 10.3389/fonc.2023.1259331

**Published:** 2023-09-28

**Authors:** Fubin Zhu, Yidan Wu, Hua Wang

**Affiliations:** ^1^ Department of Cancer Center, Chengdu Seventh People's Hospital (Affiliated Cancer Hospital of Chengdu Medical College), Chengdu, China; ^2^ Center for Geriatric Medicine Assessment and Treatment, The Fourth People's Hospital of Chengdu, Chengdu, China; ^3^ Department of General Surgery, Chengdu Public Health Clinical Medical Center, Sichuan, Chengdu, China

**Keywords:** nasopharyngeal carcinoma, concurrent chemoradiotherapy, chemotherapy, Platinum, Cisplatin

## Abstract

Nasopharyngeal carcinoma (NPC) is a malignant tumor characterized by the malignant transformation of nasopharyngeal epithelial cells. It is highly sensitive to radiation therapy, making radiotherapy the primary treatment modality. However, 60-80% of patients are initially diagnosed with locally advanced NPC (LA-NPC), where radiotherapy alone often fails to achieve desirable outcomes. Therefore, combining radiotherapy with chemotherapy has emerged as an effective strategy to optimize treatment for LA-NPC patients. Among the various chemotherapy regimens, concurrent chemoradiotherapy (CCRT) using platinum-based drugs has been established as the most commonly utilized approach for LA-NPC patients. The extensive utilization of platinum drugs in clinical settings underscores their therapeutic potential and emphasizes ongoing efforts in the development of novel platinum-based complexes for anticancer therapy. The aim of this review is to elucidate the remarkable advances made in the field of platinum-based therapies for nasopharyngeal carcinoma, emphasizing their transformative impact on patient prognosis.

## 1 Introduction

Nasopharyngeal carcinoma (NPC) is a prevalent malignant epithelial tumor, with a high incidence in Southern China and Southeast Asia. It is closely linked to Epstein-Barr virus infection, further underscoring the need for effective therapeutic interventions (1). Due to the intricate anatomical location and complex structures involved, surgical access has been limited in the treatment of NPC. Consequently, radiation therapy has remained the primary treatment modality for this type of cancer, owing to its high sensitivity to radiation. In the era of conventional two-dimensional radiotherapy (2D-RT), early-stage NPC patients achieved favorable outcomes with radical radiotherapy alone, yielded 5-year overall survival rates ranging from 86% to 97% (2–4). However, 60-80% of patients were initially diagnosed with locally advanced NPC (LA-NPC), which is associated with a higher risk of local-regional recurrence and distant metastasis (5). For these patients with locally advanced disease, radical radiation therapy alone yielded 5-year overall survival rates of 58% to 77%, with a significant propensity for distant metastasis as the primary cause of treatment failure (6–9).

Over the past few decades, photon-based radiotherapy techniques have evolved from conventional radiotherapy to three-dimensional conformal radiotherapy (3D-CRT), and subsequently to intensity-modulated radiotherapy (IMRT). Technological advancements and equipment updates have enhanced the dosimetric characteristics of radiation, resulting in improved local control and survival rates, as well as reduced occurrence of adverse reactions in nasopharyngeal carcinoma. IMRT allows for more precise coverage of the tumor while better sparing critical organs, yielding significantly superior outcomes compared to conventional 2D-RT (10). The introduction of IMRT has led to an approximate 5-6% improvement in local control rates for locally advanced NPC (11). Currently, IMRT has become the most widely employed radiotherapy technique for the treatment of nasopharyngeal carcinoma. While radiation therapy alone has shown promise in treating early-stage NPC, locally advanced cases face significant challenges, including frequent local recurrence and distant metastasis, leading to suboptimal treatment outcomes. To overcome these hurdles, the integration of radiation therapy with chemotherapy has emerged as a powerful strategy.

Concurrent chemoradiotherapy (CCRT), utilizing platinum-based agents, has now established itself as the primary and standard therapeutic approach for locally advanced NPC. The anticancer mechanisms of platinum-based drugs primarily involve the inhibition of DNA synthesis through the activation of various signaling pathways, ultimately leading to apoptosis-mediated tumor regression (12). Capitalizing on this knowledge, extensive research efforts have been dedicated to the synthesis and evaluation of platinum-based complexes as potential antitumor agents. Clinical practice has witnessed the successful application of platinum drugs such as cisplatin, carboplatin, oxaliplatin, and nedaplatin in the management of NPC. Additionally, novel platinum drugs, including lobaplatin and nedaplatin, hold considerable promise in further optimizing treatment outcomes (13, 14).

This review aims to shed light on the remarkable progress achieved in the field of platinum-based therapy for NPC, underlining its transformative impact on patient outcomes. A comprehensive understanding of the clinical applications of platinum drugs will pave the way for future advancements, fostering the development of novel and more effective therapeutic strategies to combat this challenging disease.

## 2 The evolution of platinum-based synchronous chemotherapy in conjunction with radiation therapy

Cisplatin, a first-generation platinum-based drug, is widely recognized as one of the most extensively employed anti-tumor agents in clinical settings. Its versatility as a backbone chemotherapy drug across various malignancies has remarkably elevated patient survival rates and cure rates (15, 16). The groundbreaking Intergroup-0099 study (16) revolutionized the treatment landscape for LA-NPC patients by introducing concurrent cisplatin-based chemotherapy concomitant with radiotherapy. The study demonstrated the significant augmentation of radiotherapy efficacy through synchronous cisplatin-based chemotherapy, leading to improved patient survival outcomes. This seminal research has become a cornerstone in the establishment of the prevailing standard of care for LA-NPC. In the concurrent chemoradiotherapy group, patients received regular 100 mg/m2 doses of synchronized cisplatin chemotherapy at three-week intervals during radiotherapy. CCRT substantially enhanced local control rates among LA-NPC patients and markedly improved the 3-year overall survival (OS) rate compared to radiotherapy alone (76% vs. 46%, p < 0.001). Further analysis of updated reports revealed a strikingly significant difference in 5-year survival outcomes between the two study groups: the CCRT group exhibited a robust rate of 67%, while the radiotherapy alone group only achieved 37% (p = 0.001) (17). These results have been reaffirmed through subsequent large-scale phase III clinical trials (18–23), with long-term survival data coinciding with the 10-year follow-up (24), further highlighting the superior efficacy of CCRT over radiotherapy alone. Additionally, non-randomized controlled studies (25–28) have consistently reported the advantageous therapeutic effect of CCRT compared to radiotherapy alone. Altogether, the integration of synchronous cisplatin-based chemotherapy has significantly enhanced long-term survival outcomes for patients, bestowing valuable survival benefits upon those diagnosed with LA-NPC and solidifying its position as a fundamental cornerstone within the standard treatment paradigm for this condition.

## 3 Synchronous cisplatin chemotherapy

### 3.1 Choice of chemotherapy regimen and dosage for synchronous cisplatin monotherapy

In clinical practice, chemotherapy can cause both short-term and long-term toxicity, making it challenging for patients to tolerate high-intensity synchronous chemoradiotherapy. Studies have reported that approximately 29%-48% of patients are unable to complete the full three-cycle synchronous cisplatin chemotherapy (18, 20, 29). Determining the optimal regimen and dosage of cisplatin in combination with radiotherapy for synchronous chemotherapy remains a topic of debate, influenced by factors such as toxic reactions, patient preferences, and physician expertise. In the era of conventional radiotherapy (2D radiotherapy), the dosage of cisplatin administered during radiotherapy plays a crucial role in the prognosis of LA-NPC patients undergoing only CCRT. Retrospective studies (29–33) have previously suggested that administering a synchronous cisplatin dosage of 200mg/m2 during radiotherapy can provide survival benefits for patients. Recently, a prospective clinical study (34) demonstrated promising survival outcomes in low-risk LA-NPC patients (EBV-DNA < 4000 copies/ml) treated with 200mg/m2 cisplatin administration during radiotherapy, achieving an outstanding 3-year progression-free survival (PFS) rate of 88%. Thus, there seems to be a consensus on administering cisplatin at a dosage of ≥200mg/m2 during radiotherapy.

During concurrent radiotherapy and cisplatin administration, two common approaches are used: the weekly dosing regimen and the three-week dosing regimen. The weekly dosing regimen involves administering synchronous cisplatin at a dose of 30-40mg/m2 weekly during the course of radiotherapy, while the three-week dosing regimen entails administering synchronous cisplatin at a dose of 80-100mg/m2 every three weeks. A review of the literature indicates that the survival outcomes between the two regimens are similar (32, 35–39). The 5-year OS, disease-free survival (DFS), locoregional recurrence-free survival (LRRFS), and distant metastasis-free survival (DMFS) rates for the three-week regimen range from 85.2% to 91%, 63.8% to 92.6%, 92.0% to 96.7%, and 76.1% to 95.6%, respectively. For the weekly regimen, the respective rates range from 68.9% to 96.7%, 64.9% to 90.7%, 91.0% to 96.3%, and 80.1% to 96.7% (**Table 1**). Common grade 3-4 adverse events during treatment include anemia, thrombocytopenia, leukopenia, gastrointestinal reactions (such as nausea and vomiting), and mucositis. Among the five studies that reported the incidence of adverse events, four (35, 36, 38, 39) found no significant difference in grade 3-4 adverse events between the two groups. Only one study reported a lower incidence of grade 3-4 mucositis and nausea/vomiting in the weekly regimen group but a higher incidence of thrombocytopenia compared to the three-week regimen group. However, due to the limitations of retrospective studies, larger prospective phase III clinical trials are required to further validate the current research findings. Additionally, the three-week cisplatin schedule offers convenience in terms of administration frequency compared to weekly cisplatin during radiotherapy, potentially reducing hospitalization time. Consequently, the three-week regimen is often preferred in clinical practice.

**Table 1:** Clinical studies comparing three-week regimens with single-week regimens of simultaneous cisplatin chemotherapy in LA-NPC.

Author	Year	Study design	Group	Number of cases	Stage	AJCC/UICC stage	dosages	Radiotherapy	Follow-up time (year)	OS	DFS	LRRFS	DMFS
Tao (35)	2014	retrospective	3 weeks vs. 1 week	154	II-IVb	7th	80 mg/m^2^; 30-40 mg/m^2^	IMRT	5	85.2% vs. 78.9% (P = 0.318)	71.6% vs. 71.0% (P=0.847),	93.5% vs. 92.6% (P = 0.904)	80.9% vs. 80.1% (P = 0.925)
Lee (32)	2016	prospective	3 weeks vs. 1 week	109	II-IVb	5th	40 mg/m^2^; 100mg/m^2^	3D-CRT/IMRT	3	91.0% vs. 90.8% (P=0.900)	63.8% vs. 64.9% (P=0.074)	/	/
Meng (22)	2018	retrospective	3 weeks vs. 1 week	241	III-IVb	7th	80 mg/m^2^; 30-40 mg/m^2^	IMRT	5	90.0 vs. 85.6% (P= 0.207)	92.6% vs. 85.6% (P=0.152)	96.7% vs. 94.4% (P=0.411)	95.6% vs. 88.9% (P=0.107)
Zhu (37)	2018	retrospective	3 weeks vs. 1 week	859	III-IVb	7th	100 mg/m^2^; 40 mg/m^2^	IMRT	5	91% vs. 89% (P=0.715)	81% vs. 82% (P=0.326)	92% vs. 91% (P=0.932)	91% vs. 96% (P=0.028)
Wang (38)	2019	retrospective	3 weeks vs. 1 week	322	I-IVa	8th	80-100mg/m^2^; 30-40 mg/m2	IMRT	5	88.3% vs. 96.7% (P=0.036)	80.5% vs. 90.7% (P=0.028)	93.5% vs. 96.3% (P=0.251)	91.4% vs. 96.7% (P=0.101)
Gundog (39)	2020	retrospective	3 weeks vs. 1 week	98	II-Iva	8th	100 mg/m^2^; 50 mg/m^2^	3D-CRT/IMRT	5	90.3% vs. 68.9% (P=0.11)	/	/	76.1%vs. 80.1% (P=0.74)

LA-NPC, locally advanced nasopharyngeal carcinoma; 3D-CRT, three-dimensional conformal radiation therapy; IMRT, intensity-modulated radiotherapy; OS, overall survival; DFS, disease-free survival; LRRFS, locoregional relapse-free survival; DMFS, distant metastasis free survival.

However, CCRT alone may not provide sufficient therapeutic intensity for LA-NPC patients with high-risk factors. Results from several large phase III clinical studies have confirmed the clinical importance of adding induction chemotherapy (IC) to cisplatin-based CCRT for early eradication of distant microscopic metastatic lesions, improvement of distant tumor control rates, and enhanced survival (40–44). A study found that in the era of 3D-CRT and IMRT, IC greatly reduced tumor volume, and clinical complete remission was observed in 11.3% of patients and clinical partial remission in 79.6% of patients (42). The NCCN guidelines also recommend that IC followed by CCRT as the standard treatment for LA-NPC. For patients with NPC undergoing IC followed by CCRT, several studies have suggested that a synchronous cisplatin dose exceeding 200mg/m^2^ can yield survival benefits (45–47). However, divergent conclusions have been reported in other studies. Peng et al.’s research (47) indicated that a dose of less than 200mg/m^2^ during CCRT following IC can enhance patients’ 4-year overall survival (OS) and distant metastasis-free survival (DMFS). Conversely, Lv et al.’s study (48) proposed that there is no significant difference in survival outcomes between patients receiving a cisplatin concurrent dose of ≥200 mg/m^2^ and those receiving <200 mg/m^2^. Further validation through large-scale Phase III clinical trials is still warranted.

### 3.2 Synchronous chemotherapy with cisplatin in combination with other drugs

During radiotherapy, synchronous chemotherapy regimens based on cisplatin, in combination with two or three drugs, have been continuously explored for LA-NPC. The most common regimen is fluorouracil combined with cisplatin (PF) (49), and other studies have also investigated the safety and efficacy of regimens including docetaxel, fluorouracil combined with cisplatin (TPF) (50), TP regimen (51–53), and gemcitabine combined with cisplatin (GP) (54, 55). Furthermore, studies have reported on the efficacy of raltitrexed plus cisplatin (56), cetuximab plus cisplatin (57, 58), and nimotuzumab plus cisplatin (14, 59, 60). Overall, the addition of chemotherapy or targeted drugs to cisplatin-based synchronous chemotherapy did not increase efficacy compared to cisplatin alone. In addition, some uncommon single-agent synchronous chemotherapy regimens have been reported for the treatment of nasopharyngeal carcinoma, including paclitaxel (61), S-1 (47, 48, 62, 63), cetuximab (64), and nimotuzumab (65), but they did not demonstrate superior efficacy or lower toxicity compared to single-agent cisplatin.

Firstly, the relatively small sample sizes of these studies may result in insufficient statistical power and potential selection bias, thereby compromising the robustness of the research findings. Secondly, some studies are retrospective in nature and predominantly conducted within a single center, necessitating multicenter, prospective, large-scale randomized clinical trials to further investigate the efficacy of these regimens. Currently, these approaches lack substantial research evidence to support their use. Overall, the synchronous chemotherapy combining cisplatin with other drugs does not appear to enhance efficacy; instead, it may lead to more severe hematological or non-hematological toxicities.

## 4 Synchronous chemotherapy with other platinum agents

Cisplatin-based synchronous chemotherapy regimens are associated with increased acute and late toxicities during radiotherapy, including severe gastrointestinal reactions (such as nausea and vomiting), renal toxicity (66), and ototoxicity (67, 68), posing limitations to the use of cisplatin. Furthermore, some patients develop resistance to cisplatin, which reduces its effectiveness, particularly when tumors recur (69). As a result, there is a growing demand for other platinum-based chemotherapy agents that can provide similar efficacy to NPC but with fewer side effects. Platinum derivatives such as nedaplatin, lobaplatin, and carboplatin have been explored as alternatives to cisplatin for the treatment of NPC.

### 4.1 Carboplatin

Carboplatin, a second-generation platinum agent, is also utilized for the treatment of various malignancies. A small retrospective study involving 75 LA-NPC patients (70) demonstrated poorer 3-year survival outcomes in the group receiving 2 cycles of synchronous carboplatin chemotherapy. A non-inferiority clinical trial (71) comparing the efficacy of cisplatin and carboplatin in synchronous chemoradiotherapy for LA-NPC revealed no significant differences in 3-year overall survival (OS; P=0.98) and disease-free survival (DFS; P=0.96) between the synchronous carboplatin and cisplatin groups. However, data from their latest multicenter study (72) indicated that adding adjuvant chemotherapy to carboplatin-based synchronous chemoradiotherapy did not significantly improve short-term efficacy but increased toxicity. Another phase II clinical trial (73) demonstrated favorable outcomes with carboplatin-based synchronous chemoradiotherapy for LA-NPC, with a 3-year OS rate of 83.6% and a PFS rate of 65.3%. Additionally, patients exhibited good compliance. Nevertheless, there is still controversy regarding the evidence supporting the equivalence of second-generation platinum agent carboplatin to cisplatin.

### 4.2 Nedaplatin

Nedaplatin is a cisplatin analog with similar antitumor mechanisms and therapeutic effects, but it does not require hydration to protect the kidneys. Several studies (74–76) have compared the efficacy of nedaplatin and cisplatin in synchronous chemoradiotherapy, suggesting that nedaplatin may be a promising alternative to cisplatin, as it is effective and safe for treating NPC. The results of a randomized Phase III controlled trial (77) indicated that for stage II-IVB NPC patients, nedaplatin-based CCRT is not inferior to cisplatin-based CCRT in terms of the 2-year progression-free survival (PFS). Moreover, the cisplatin group had a higher incidence of Grade 3-4 adverse events. The recently updated 5-year follow-up results (78) support the initial findings. Additionally, from a cost-effectiveness analysis perspective, nedaplatin-based synchronous chemoradiotherapy holds an advantage (79). Overall, nedaplatin appears to be one of the potential alternatives to cisplatin in synchronous chemoradiotherapy for LA-NPC. Ongoing studies such as NCT04472403, NCT01479504, NCT01265147, NCT04437329, and NCT03503136 are further evaluating the efficacy of nedaplatin in NPC, and there are also ongoing explorations of combination regimens involving nedaplatin (80, 81).

### 4.3 Lobaplatin

Lobaplatin is a third-generation platinum agent. Previous studies have reported that lobaplatin can overcome certain forms of resistance caused by other platinum agents (82). The results of a Phase II trial (83) validated the efficacy and safety of lobaplatin-based induction chemotherapy followed by CCRT in the treatment of LA-NPC. Subsequently, the results of a large Phase III randomized non-inferiority controlled trial (84) demonstrated that lobaplatin-based induction chemotherapy plus CCRT for LA-NPC had similar survival outcomes and side effects compared to cisplatin-based treatment. A subsequent commentary (85) indicated that lobaplatin is not inferior to cisplatin and has lower toxicity, making it a promising alternative to cisplatin. Additionally, ongoing clinical studies such as NCT04472403, NCT03196869, ChiCTR1900021536, and ChiCTR-IIR-17013112 aim to further evaluate the benefits and risks of lobaplatin in nasopharyngeal carcinoma and validate the value of these treatment strategies (**Table 2**).

**Table 2:** Ongoing clinical trials on platinum drugs in LA-NPC.

Agent	Condition	Phase	Study type	Recruiting status	Locations	treatment	Intervention	Enrollment(estimated)	NCT/ChiCTR1 no.
Cisplatin									
	Low-risk LA-NPC	III	Interventional	Recruiting	China	CCRT	P-RT vs. Nimotuzumab-RT	36	NCT04456322
	NPC with stage T1-4N2-3 or T3-4N0-1M0	III	Interventional	Recruiting	China	IC+CCRT	P-RT vs. GP +RT alone	236	NCT02460887
	LA-NPC	II	Interventional	Recruiting	China	CCRT	P-RT vs. TP-RT	164	NCT03047265
	NPC with stage II-III	III	Interventional	Recruiting	China	CCRT	DDP-RT vs. Nimotuzumab-RT	384	NCT03837808
	NPC with stage II-IVA	II	Interventional	Recruiting	China	CCRT	P-RT vs. P+ Nimotuzumab-RT	246	NCT04223024
	LA-NPC	III	Interventional	Recruiting	China	CCRT	P-RT vs. RT alone	440	NCT03015727
Lobaplatin									
	LA-NPC	II	Interventional	Recruiting	China	IC+CCRT	DLF+L-RT vs. DPF+P-RT	128	NCT03196869
	LA-NPC and low-risk NPC	III	Interventional	Recruiting	China	CCRT	L-RT vs. P-RT	434	ChiCTR1900021536
	LA-NPC	II	Interventional	Recruiting	China	IC+CCRT	DL+L-RT vs. DP+ P-RT	120	ChiCTR-IIR-17013112
Nedaplatin									
	LA-NPC	II	Interventional	Recruiting	China	CCRT	N-RT vs. P-RT	20	NCT01265147
	LA-NPC	III	Interventional	Recruiting	China	IC+CCRT	DNF+P-RT vs. DPF+P-RT	352	NCT04437329
	LA-NPC	III	Interventional	Recruiting	China	IC+CCRT	DNF+N-RT vs. DPF+P-RT	632	NCT03503136
	LA-NPC	III	Interventional	Recruiting	China	IC+CCRT	DP+ P-RT vs. DN+N-RT	NA	NCT01479504
Carboplatin									
	NPC with stage T3-4NxM0 or TxN2-3M0	III	Interventional	Recruiting	China	IC+CCRT	DC+C-RT vs. DP+P-RT	482	NCT03919552
	LA-NPC	II/III	Interventional	Recruiting	China	IC+CCRT	TGC+P-RT vs. P-RT	172	NCT00997906

NPC, nasopharyngeal carcinoma; LA-NPC, locally advanced nasopharyngeal carcinoma; IC, induction chemotherapy; RT, radiotherapy; CCRT, concurrent chemoradiotherapy; P, cisplatin; L, lobaplatin; N, nedaplatin; C, carboplatin; G, gemcitabine; T, paclitaxel; D, docetaxel; GP, gemcitabine, and cisplatin; TP, paclitaxel and cisplatin; DP, docetaxel and cisplatin; DC, docetaxel and carboplatin; DL, docetaxel and lobaplatin; DNF, docetaxel, nedaplatin, and fluorouracil, DPF docetaxel, cisplatin, and fluorouracil, DLF, docetaxel, lobaplatin, and fluorouracil.

### 4.4 Oxaliplatin

A phase III clinical study (86) explored the efficacy of oxaliplatin monotherapy combined with synchronous chemotherapy compared to radiation therapy alone. The findings indicated that the oxaliplatin group demonstrated a more favorable short-term survival profile; however, extensive randomized trials are warranted to thoroughly evaluate its comparative effectiveness against cisplatin.

## 5 Platinum and sequential treatment approach

The results of several large phase III clinical studies confirm that the addition of cisplatin-based induction chemotherapy (IC) to cisplatin-based CCRT is important for the early eradication of distant microscopic metastatic lesions, the improvement of distant tumor control and the enhancement of survival (40, 41, 43, 44, 47). The administration of 2-4 cycles of IC followed by CCRT has been shown to increase treatment-related toxicity and hinder patients’ ability to withstand subsequent high-intensity CRRT. Studies have reported that following IC, approximately 8% to 13% of patients do not complete the intended two cycles of synchronous cisplatin chemotherapy, while 22% to 39% do not complete three cycles (100mg/m2 cisplatin every three weeks) of CCRT (40, 44, 87). In addition, NPC patients receiving IC plus CRRT treatment exhibit higher rates of grade 3 or 4 adverse events compared to those receiving CRRT alone, with 20% to 40% of patients unable to complete the originally planned course of synchronous radiotherapy due to severe toxicity (44, 87, 88). However, interruptions and extensions of radiotherapy have been shown to have detrimental effects on patient survival (89). The dosage of cisplatin administered during concurrent chemoradiotherapy (CCRT-DDP) is a significant prognostic factor for LA-NPC patients, and a dose of 200mg/m2 of synchronous cisplatin may already be deemed adequate (29, 30, 34, 46, 47). While induction chemotherapy is also cisplatin-based, there is currently a lack of research focused on examining the association between the dosage of cisplatin administered during the entire treatment course and the survival outcomes of LA-NPC patients receiving IC followed by CCRT. While there is currently a lack of investigation on the therapeutic efficacy of other platinum-based drug dosages in LA-NPC patients, regarding lobaplatin and nedaplatin, significant research is warranted. In terms of toxicity reactions, it appears that lobaplatin and nedaplatin may offer a potential reduction in severe acute adverse effects compared to cisplatin. However, further exploration is required through robust head-to-head large-scale studies. Currently, there is a dearth of research exploring the impact of platinum-based drug dosages on the therapeutic efficacy of lobaplatin and nedaplatin in LA-NPC patients. However, concerning toxicity reactions, it appears that both lobaplatin and nedaplatin might offer a potential reduction in severe acute adverse effects compared to cisplatin (90). Nevertheless, further investigation is required through extensive head-to-head studies with large sample sizes to validate the finding.

## 6 Conclusions and prospects

In regard to the treatment of NPC, platinum-based chemotherapy agents play a pivotal role. At present, platinum-based concurrent chemoradiotherapy, particularly cisplatin-based regimens, stands as the standard treatment for NPC. Nevertheless, investigations have revealed that combining cisplatin with other drugs in synchronous chemotherapy fails to enhance overall survival rates and may, in fact, increase the incidence of adverse reactions. Consequently, further research is imperative to elucidate the optimal dosage and regimen for cisplatin monotherapy in synchronous chemotherapy protocols for NPC. Furthermore, while there may be no substantial disparities in efficacy and toxicity between weekly and three-week regimens, the latter offers improved convenience and reduced hospitalization duration.

Distinct species of platinum-based chemotherapeutic agents possess individual merits. In an ideal scenario, alternative platinum agents should be able to replace cisplatin while boasting comparable activity, efficacy, and decreased toxicity. However, debates regarding the equivalence of second-generation platinum salts, such as carboplatin, to cisplatin in NPC patients remain unresolved. Nedaplatin, with its advantageous low cost and capacity to serve as a substitute for cisplatin-resistant patients or those intolerant to cisplatin’s side effects, represents a prospective alternative. Notably, lobaplatin, a novel generation platinum derivative, has exhibited remarkable efficacy in the management of NPC, matching cisplatin in therapeutic outcomes while demonstrating a lower incidence of toxic reactions. Nonetheless, due to its higher price compared to cisplatin, the inclusion of lobaplatin in medical insurance coverage could potentially mitigate the economic burden on patients. Lobaplatin and nedaplatin may serve as compelling avenues for advancing research in upgrading synchronous chemoradiotherapy strategies for locally advanced NPC. However, when substituting platinum salts, further study is crucial to explore alternative individualized treatment strategies, including administration dosage and regimens, aimed at alleviating long-term toxic reactions and economic burdens faced by LA-NPC patients.

Furthermore, there is ongoing exploration of platinum-based chemotherapy combined with immune checkpoint inhibitors as a first-line treatment strategy for recurrent/metastatic NPC, demonstrating notable advantages (91–93). In the case of locally advanced NPC, several ongoing clinical trials, including NCT03700476, NCT04557020, NCT04447612, NCT04447326, NCT04782765, and NCT03734809, are continuously investigating the potential advantages of integrating immunotherapy into platinum-based IC. This emerging combination holds significant potential as a prospective therapeutic option for the future management of LA-NPC.

## Author contributions

FZ: Conceptualization, Methodology, Writing – original draft, Writing – review & editing. YW: Conceptualization, Methodology, Writing – original draft, Writing – review & editing. HW: Conceptualization, Writing – review & editing.
